# Reliability and validity of an adapted hip abductor strength measure as a potential new fall risk assessment for older persons: a study protocol

**DOI:** 10.1186/s12877-021-02048-6

**Published:** 2021-02-05

**Authors:** Simone Chantal Gafner, Caroline Henrice Germaine Bastiaenen, Emmanuel Biver, Serge Ferrari, Lara Allet

**Affiliations:** 1 Geneva School of Health Sciences, HES-SO University of Applied Sciences and Arts Western Switzerland, Geneva, Switzerland; 2 Department of Epidemiology, Research Line Functioning, Participation and Rehabilitation, CAPHRI, Maastricht University, Maastricht, the Netherlands; 3 Division of Bone Diseases, Department of Medicine, Geneva University Hospitals and Faculty of Medicine, University of Geneva, Geneva, Switzerland; 4 School of Health Sciences, HES-SO Valais-Wallis, University of Applied Sciences and Arts Western Switzerland, Valais, Switzerland; 5 Department of Medicine, Geneva University Hospitals and Faculty of Medicine, University of Geneva, Geneva, Switzerland

## Abstract

**Background:**

Persons aged ≥ 65 years are currently the world’s fastest growing age group. An important complication of age is the increasing risk of falls. Falls have multifactorial etiology and modifiable risk factors open for interventions in prevention and rehabilitation, are of high interest. In this context, strong hip abductors seem to be important to prevent falls. A newly adapted measurement device to measure hip abductor strength (HAS) in a closed chain position was developed. We aim to assess feasibility, intra- and inter-tester reliability and construct and criterion validity of the new measure.

**Methods:**

In two subsequent parts a feasibility, reliability and validity study with an adapted measurement instrument for the assessment of HAS (index test) in a closed chain position in persons aged ≥ 65 years will be conducted. Part I investigates feasibility of the measure in clinical settings as well as reliability of the new HAS test (*n* = 26). Part II evaluates construct and criterion validity (*n* = 169). Construct validity will be assessed cross-sectional, criterion validity by comparison with prospectively followed up fall history for 12 months (external criterion) and other functional fall risk assessments (Short Physical Performance Battery, Timed Up and Go test, usual gait speed and hand grip strength).

**Discussion:**

Results of feasibility, will give insight in its applicability in daily clinical life and clinimetric properties will show if measurements of HAS in a closed chain position should be encouraged to include in fall risk assessments in older adults.

## Background

Persons aged ≥ 65 years are currently the world’s fastest-growing age group [1]. The United Nations state that one in six persons in the world and one in four persons living in Europe and Northern America will be aged over 65 by 2050 [1]. They also predict that the number of persons over 80 years is going to triple, from 143 million in 2019 to 426 million in 2050 [1].

An important complication of age is the increased risk of falls. About one-third of community dwellings over 65 years and up to half of those in long-term care institutions fall at least once a year [2]. Those impressive numbers show that fall risk detection and prevention is a global public health challenge and should have high priority. Falls can cause serious consequences such as loss of independence [3] and increased mortality [4]. Additionally to a fall itself, older persons are in a high risk to enter in a vicious cycle of deconditioning after a fall [5]. A first fall and related reduced physical conditions combined with fear of falling might lead to insecurity and to even more falls [5]. It is of utmost importance older persons at risk of falls could be detected as soon as possible, to support them in preventing falls and postponing recurrent falls with the aim to reinforce them to reduce serious personal consequences.

Falls have a multifactorial etiology and risk factors can be categorized in environmental, psychological, or cognitive factors, as others linked to medication and physiological aspects [6]. Of high interest are modifiable risk factors open for interventions in prevention and rehabilitation. Compromised mobility, balance as well as diminished strength of the lower extremities are within this context mentioned in a systematic review of Sousa et al.[6].

Functional lower-extremity weakness has been shown important to enhance the risk of the transition from a non-faller into a faller [7]. Hip abductor muscle strength shows a more pronounced decline in older persons than hip extensor muscle strength of the leg [8]. The hip abductor muscle group particularly also is perceived to be important for older persons because of their significant role in maintaining mediolateral balance control [9–11] and their relation to lateral and posterolateral falls [12]. Furthermore, hip frontal plane muscle strength and spinal moments of force are important parameters to counteract destabilization in the mediolateral direction during single-leg support and stabilizing head, arms and trunk over the support leg and thus prevent the center of mass to rapidly fall downward and lateral toward the unsupported swing side [13]. At the actual moment of impact of an acute fall, muscle contraction of the hip abductors also seems to act protectively against stress-induced on the femoral neck and likewise the risk of a femoral fracture [14].

In our previous research about hip abductors strength and fall risk of older persons, we found that hip abductors strength measured in a side-lying position of the participant can discriminate quite well between older fallers and non-fallers [15–17]. However, the hip abductor strength assessment procedure used in these studies was too long (mean 10.58 ± 1.56 min ) for clinical use [16]. A side-lying position also is not functional as falls usually occur during walking and climbing stairs [7]. Measuring hip abductor strength (HAS) in an adapted position; in a standing, closed chain position in which the hip abductors must help stabilize the upper body over the single-leg support and prevent the lateral shift of the center of mass towards the swing side [13], seems much more functional and therefore interesting.

We developed an adapted measurement device, based on our experiences with our already existing measure in a side-lying position, to measure HAS in a closed chain position.

The objective of the present study is to assess feasibility, intra-, and inter-tester reliability as well as construct and criterion validity of the adapted measure in a standing, closed chain position. If reliable and valid this measure would offer caregivers easily feasible possibilities to assess HAS in a functional position in a daily clinical setting. The inclusion of hip abductors strength in the multifactorial assessment of the risk of falls might help to improve the detection of persons at risk of falls and to shape a well targeted guidance for those persons. Benefits will concern a substantial percentage (~ 40 %) [2] of the permanently growing total number of the population aged ≥ 65 years.

## Methods and analysis

A feasibility, reliability, and validity study with a new measurement instrument for the assessment of hip abductor strength (index test) in a closed chain position (described below), in persons aged ≥ 65 years will be conducted in two subsequent parts.

Part I: Feasibility of the measure in clinical settings as well as the intra- and inter-tester reliability of the new hip abductor strength test will be assessed.

Part II: Construct and criterion validity of the hip abductor strength values, assessed with the new measurement instrument, will be evaluated.

**Fig. 1:**
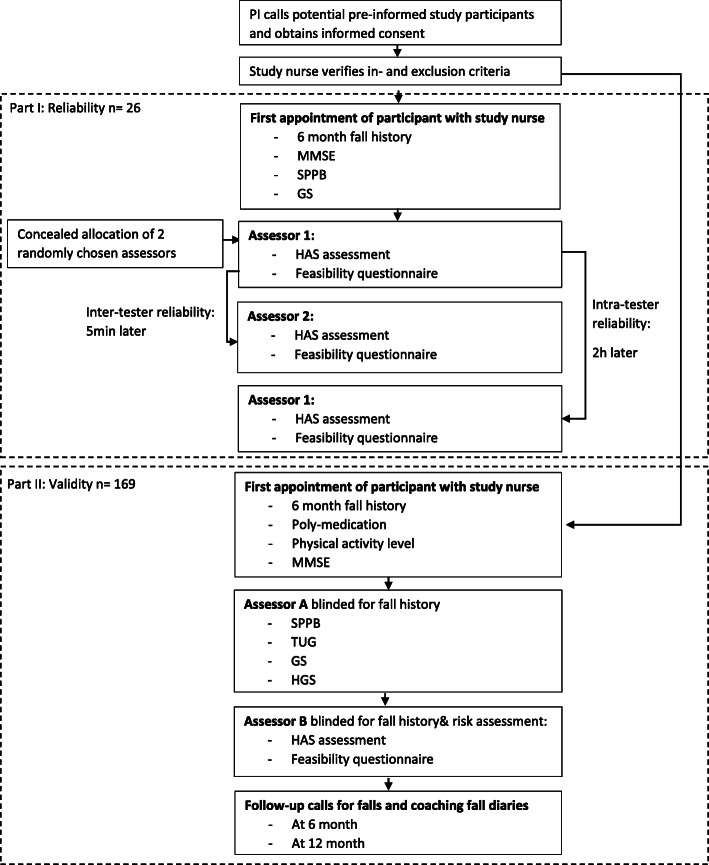
Flow of participants through the study. PI Principal Investigator, MMSE Mini Mental State Examination, SPPB Short Physical Performance Battery, GS Gait Speed, HAS Hip Abductor Strength, TUG Timed Up and Go, HGS Hand Grip Strength

The presented study protocol is a clinimetric study nested within a prospective cohort study about an integrative fragility fracture prediction model called AFFIRM-CT study, including participants aged ≥ 65 years. The protocol follows the SPIRIT guidelines, which are an international standard for trial protocols and provide recommendations for their content [18]. Participants flow through the study (part I and part II) is shown in Fig. 1.

### The index test; description of the hip abductors strength test used in part I and II

**Fig. 2:**
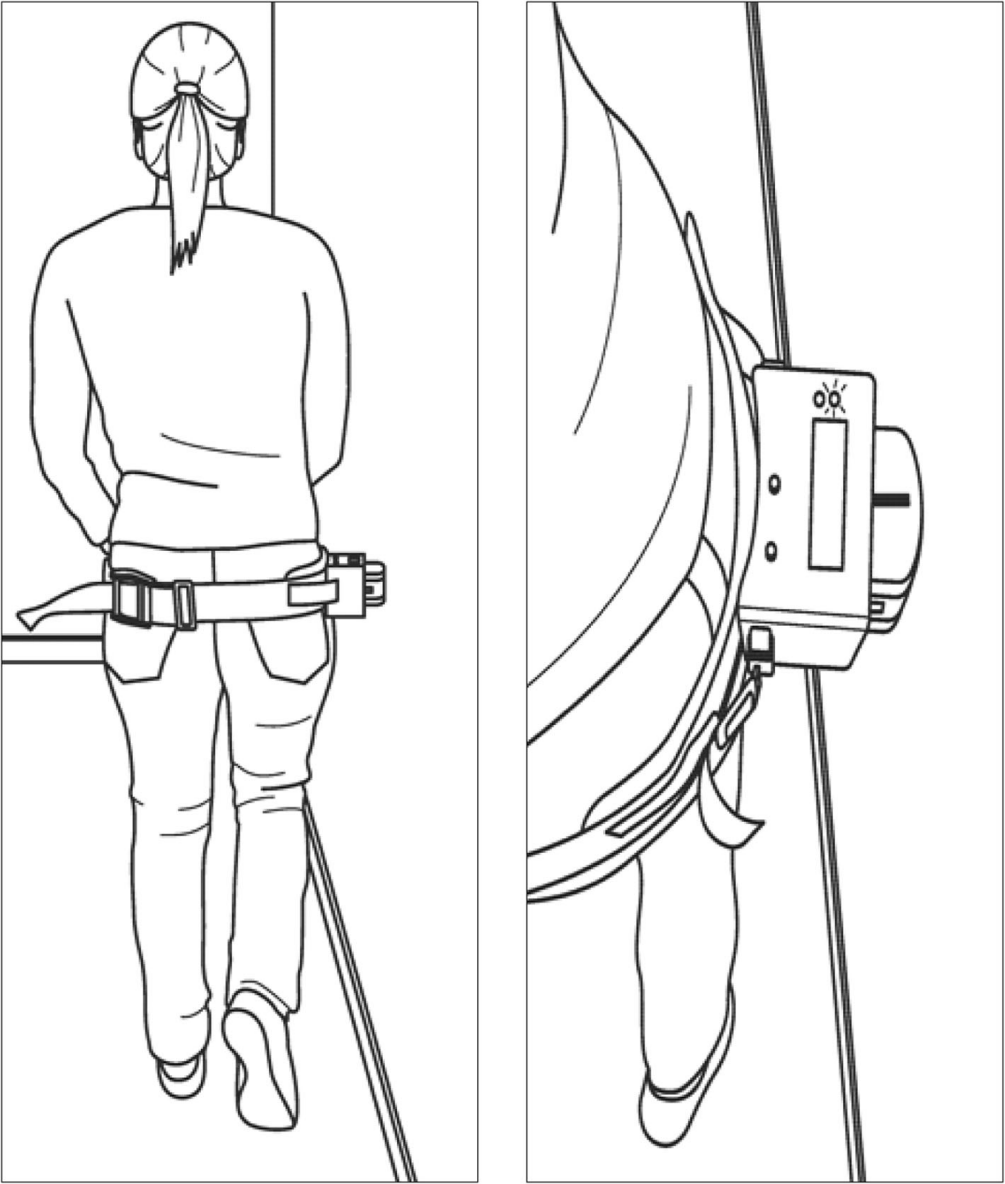
Schematic picture of our measurement instrument and our testing position during the measurement procedure

A force measurement device will be attached to the participant with a belt on the height of the great trochanter of the non-tested leg. The participant will be invited to stand sideways close to a wall, with their hip in a neutral position in the sagittal and frontal plane (Fig. 2). The patient’s shoulder should not touch the wall. A chair for stabilization will be placed in front of the tested person.

The participant is invited to raise the non-tested leg (the leg close to the wall) up and flex the knee approximately 20°, in a way that the foot has no floor contact (Fig. 2). The tester stands behind the participant and guides the participant’s pelvis in direction of the wall by tactile and verbal information until the dynamometer is slightly touching the wall (correct starting position).

The participant then is asked to push with the standing leg (the one further away from the wall) as hard (assessment of the maximum voluntary isometric contraction (MVIS)) and in the second round as fast as possible (assessment of the rate of force generation (RFG)) in the direction of the wall to squeeze the dynamometer between the pelvis and the wall. Verbal encouragement will be given by saying to “push”, “push”, “push”, … against the wall over a five second time span. One to three submaximal test trials are used until the participant understands the test movement. The test is repeated three times per leg (6 trials in total for the assessment of MVIS and 6 trials for the RFG of both legs, with a break of 30 s between each trial).

### Part I: Intra- and inter‐tester reliability and feasibility

Reliability, in the context of this study, is « the extent to which scores for patients who have not changed (between the subsequent test performances) are the same for repeated measurements under several conditions: e.g. by different persons on the same occasion (inter-tester); or by the same persons on different occasions (intra-tester)» [19].

#### Study aims and hypotheses

The aims of part I are the evaluation of the intra- and inter-tester reliability of the results of the measurements, by health care professionals. According to our previous studies, we hypothesize that the intra- and inter-tester reliability will achieve ICC_agreement_ values of 0.8 and above, corresponding to good to excellent reliability [20]. We further aim to list some aspects of feasibility of the new testing procedure. We will list if the testing procedure is well tolerated and appreciated by the participants and can be performed in a feasible time frame for clinical use (< 10min).

#### Study design

Part I is a reliability study within a cross-sectional design. It is the first study evaluating the reliability of the new testing procedure and an important first step for a later implementation in daily clinical use.

#### Participants in and exclusion criteria

Participants aged ≥ 65 years, able to walk household distances with or without an assistive device, hospitalized, already participating in the AFFIRM-CT study, and have signed informed consent for this part will be included. We excluded participants with cognitive, orthopedic, or neurological disorders associated with an increased fall risk (e.g. moderate or severe dementia (MMSE < 18) [21], participants with stroke, persons with Parkinson’s disease, persons who had a hip replacement within the previous year) [6, 22] and persons who presented contraindications for or would adversely affect the strength tests (see our previous publication [17]) .

#### Procedure

At the moment of enrollment between January 2021 and January 2023, participants of the cohort of the AFFIRM-CT study will be asked to participate in this nested-in study. The study nurse of the cohort will verbally inform potential study participants about this study and distribute written information. One week after having consented to the study nurse to be approached for the study, the principal investigator (PI) will call the potential study participants to ask if they have questions, if they agree to participate (hand-in the signed informed consent) and give their permission to forward already collected relevant participant information collected within the cohort towards the nested-in study. As soon as the PI obtains the informed consent, the study nurse will consult the participants’ information and check the respective in- and exclusion criteria (described above) for the designated participant. When he can be included, the study nurse will arrange the first appointment. For the diagram of participants flow through the study, the study nurse will record the characteristics of all interested participants in a coded form.

Socio-demographic data (age, sex, height, and weight) will be extracted from the participant record of the cohort study. In addition, participants' fall history in the last 6 months [6] and the Mini Mental State Examination questionnaire (MMSE) [21] will be assessed by the study nurse. Finally, the study nurse will ask the participant to perform the short physical performance battery test (SPPB) [23, 24] and assess her/ his usual gait speed on a 4m walkway [25, 26]. These two functional fall risk assessments are part of the relevant characteristics of the study population.

The inter-tester reliability will be evaluated between two assessors (health care professionals). The exact testing procedure for the index test, hip abductors strength assessment, is described earlier in the method section. For the evaluation of the inter-tester reliability, a concealed allocation of two randomly chosen assessors (health care professionals) will measure the hip abductors strength of a participant with a 5min break between the assessments of the two assessors (see randomization and conceal allocation section below). Similar to instructions in clinical situations, the assessors will get a short 10min instruction of how to use the dynamometer prior to the measurements. The HAS (MVIS and RFG) of the left or right side (randomly chosen) will be evaluated. The patient will be asked to push the dynamometer first as hard as possible (assessment of MVIS) and in a second-round as fast as possible against the wall (assessment of RFG). Every test (MVIS and RFG) will be repeated three times with a break of 30 s between each trial. The two assessors will not discuss the collected strength results and will be blinded to the hip abductors’ strength test results of the other assessor as well as to all fall risk related parameters (e.g. fall history, results of other functional fall risk assessments).

Participants will then have a break of two hours before the re-assessment of their HAS by the pre-designated assessor for the evaluation of the intra-tester reliability.

The intra-tester reliability will be evaluated by two measurements with the same participant and by the same assessor. The two assessments will be done two hours apart, an offset considered to be close enough so that no health status change regarding the HAS measurement is expected but far enough that the participants can recover between the two test sessions. The assessor will be blinded for all fall risk related parameters.

Directly after the two sessions, participants will be asked to answer a short “feasibility” questionnaire about their perception during the test sessions and their respective motivation to complete the tasks. For the assessment of the feasibility of the new measurement instrument, the time to complete the hip abductors strength test will be recorded in seconds for the three trials per leg (one side, MVIS, and RFG). The assessor additionally will be asked to complete a short questionnaire with multiple choice answer options and open questions about the feasibility of the test in clinical life (comprehensibility of the test instructions for the participant and practical points for the installation of the measurement device (belt, manageability of the device, starting and stopping of measurements)). Participants’ also will be asked to complete a questionnaire with closed and open question options about their perception of the test (duration of the test), in case that any pain occurred during or after the test indicate the intensity on the numerating rating scale (NRS) and indicate on a body chart where the pain occurred. Next, there will be some questions about the comprehensibility of the instructions and the test. In both questionnaires, the one for the assessors as well as the one for the participants, a field for comments will allow giving open-ended feedback on all aspects of the measurement feasibility.

#### Randomization and allocation concealment

For part I of the study, two concealed randomizations will be needed; once for the order of the assessors for the inter-tester reliability and once to decide the order of the tested leg for each patient. The concealed block randomization will be performed, in a block size of four with a validated electronic randomization system, and will be performed by a secretary, completely independent of the study and does not have access to raters nor patients. The allocation concealment will be granted by sequentially numbered, opaque, sealed envelopes one pile with white envelopes for the order of the assessors and one with grey envelopes for the order of the tested leg, which the secretary will assign just before the beginning of the testing procedure.

#### Data collection

 The data collection will be performed as soon as the participant officially gave their permission and signed the informed consent. Participants’ information and test results will be entered in the electronic case report form (e-CRF, see “Data handling and storage for part I and part II of the study” section).

##### Sociodemographic data and population characteristics

The socio-demographic data (age, sex, height, and weight) will be extracted from the electronic participant record of the AFFIRM-CT cohort study.

Participants’ fall history in the last 6 months [6] will be obtained by personal questioning with a beforehand explanation of the definition of a fall. A fall is defined as an event resulting in a person inadvertently coming to rest on the ground, floor, or other lower level [2].

Cognitive impairment is known as an influencing factor of the fall risk in older adults [27]. Consequently, the study nurse will assess cognitive impairments with the Mini Mental State Examination (MMSE), a validated assessment tool for older persons [27, 28].

##### Functional fall risk assessments

To complete the data of the participants’ characteristics the study nurse will further assess the SPPB [24] and participants’ usual gait speed on 4m [25, 26]. The SPPB is a functional physical measure of performance for older persons [29] with possible scores ranging from zero (worst) to 12 points (best). For the assessment of the walking speed, participants will be asked to twice walk a 4m distance at their usual speed with a static start [26]. The faster of the two trials will be retained for the analyses [26].

##### Index test; hip abductors strength test

The data collection for the HAS tests is described earlier in the method section. The strength values will be entered in the e-CRF.

#### Sample size calculation for the reliability of the index measure

According to previous study results assessing HAS [30] we are expecting ICC_agreement_ values of 0.8 and above, corresponding to good to excellent reliability for the intra- and inter-tester reliability [20]. For the 95 % confidence intervals, we are expecting values of ± 0.2 which lead us to a sample size of 23, calculated with the statistical environment R [31] (package: ICC sample size), for part I. Considering a 10 % drop out rate, we will include 26 participants. All 26 participants will be assessed for inter-, and intra-tester reliability.

#### Data analysis

Data analyses will be performed with SPSS version 25 (IBM Corp. Released 2017. Armonk, NY: IBM Corp.). Descriptive statistics for the sample characteristics, time to complete the tests, and number of participants able to perform the test will be performed. For continuous variables, we will present the mean and standard deviation (mean ± SD). Intra-tester and inter-tester reliability will be assessed using the ICC_agreement_ (A, 1) model, which assesses the degree of agreement among measurements assuming a two-way random-effects model [15, 32]. In addition, we will compute 95 % confidence intervals (CI) through bootstrapping (5000 resamples, bias-corrected, and accelerated percentile method) [15]. The standard error of measurement (SEM) will be computed as follows: SEM= $\surd \left({\sigma}_{observer}^2+{\sigma}_{residual}^2\right)$ where *σ*_*observer*_ is the variance between the two testers and the residual variance, which is partly due to the unique combination of participants and observers [33, 34]. The minimal detectable change (MDC) will be calculated as follows: MDC = 1.96 ∗ SEM∗ √2. The MDC will be normalized by the mean and expressed in percentage. We will further use Bland Altman analyses to investigate absolute reliability and determine between session agreements of the strength measurements [35, 36]. We will also present the 95 % limits of agreement (LoA 95 %) representing two SD above and below the mean differences between the sessions [30].

### Part II: construct and Criterion Validity

The validation will be performed according to the principles of COSMIN [34] and following the checklist of « Standards for Reporting Diagnostic accuracy studies » (STARD) [37]. The construct we are interested in, in part II of this study, is measuring the “fall risk” of persons aged ≥ 65 years. As indicated in the introduction, falls and fall risk are influenced by a vast number of influencing factors with a very multifactorial etiology (a multidimensional construct). Therefore, “fall risk” is a rather abstract construct, which researchers and clinicians try to assess indirectly by a variety of different tests. Interesting factors for prevention or rehabilitation interventions for “fall risk” are performance variables like balance and strength, which are modifiable by non-pharmacological interventions of health care professionals (e.g. physiotherapists, ergotherapists, etc.) and open to improvement. Therefore, in our field “fall risk” is currently assessed by performance tests like the SPPB [24], the timed up and go (TUG) [38], gait speed (GS) [26], as well as hand-grip strength (HGS) as a parameter, assumed to be related to whole-body strength and fall risk [39, 40]. These tests will be used to assess the construct validity as they are all assumed be measuring one or more aspects of the not directly to measure construct “fall risk”.

For the evaluation of the criterion validity, the fall history will be used as the predefined external criterion also called the reference standard or gold standard in this part of the study. It is assumed to correctly classify the participants into fallers or non-fallers. Participants not reporting a fall in the follow-up phase of the study and/or within the last 6 months will be classified as non-fallers [41]. Participants that report one or more falls will be classified as fallers [41]. Participants were informed that a fall was defined as an event resulting in a person inadvertently coming to rest on the ground, floor, or other lower level [2]. Ideally, fall history is assed in a prospective follow up period where participants can closely be followed and their number of falls regularly assessed.

#### Study aims and hypotheses

For the evaluation of construct validity of the hip abductors’ strength measurement, we will compare the hip abductors strength to other currently used functional fall risk assessments like the SPPB [24], TUG [38], GS [25], and HGS [40]. We aim to assess the strength and direction of the correlation between currently used fall risk assessments SPPB, TUG, GS, and HGS with the hip abductors strength, each separately. We hypothesize to find moderate [42] positive correlations (0.40–0.69) between hip abductors strength, SPPB, GS, and HGS and moderate negative correlations between the TUG and hip abductors strength. We are expecting moderate correlations as HAS assesses the same construct but not completely the same parameters as the other fall risk assessments. SPPB and TUG, and in some ways GS, include the assessment of functional parameters like gait and balance, whereas HGS and HAS assessment include only strength. We expect the correlations between hip abductors, SPPB, GS, and HGS to be positive and hypothesize to find the highest –correlation (close to the upper boundary of a moderate correlation ≤ 0.69) between SPPB and HAS as both include a specific strength assessment of the lower limb. The correlation between HGS and HAS is expected to be rather close to the lower boundary of a moderate correlation (≥ 0.4) as they don’t assess the strength of the same body parts. The correlation between the TUG and HAS is hypothesized to be almost as strong as between the SPPB and HAS but due to the scoring of the TUG in a negative direction.

The criterion validity will be evaluated considering HAS as the index test and fall history of the last 6 months as well as falls during the follow-up time of 12 months as the predefined reference standard. We intend to evaluate if hip abductors’ strength assessed with the new procedure is a good diagnostic tool to classify older persons into fallers and non-fallers and/or can improve fall risk detection by combining it with other functional fall risk assessments. Therefore, for the criterion validity we aim to investigate the diagnostic accuracy of HAS (MVIS and RFG), by evaluating its area under the curve [AUC], sensitivity [sens], specificity [spec], positive predictive value [PPV], negative predictive value [NPV], and positive and negative likelihood ratio [LR+, LR-] and the respective, clinically relevant cut-off values. We will compare these values, in the same population, with the indicators of diagnostic accuracy for TUG, SPPB, GS and HGS. We will further calculate net sensitivity and specificity of HAS to get insight in its possible combination with our chosen fall risk assessments (TUG, SPPB, GS and HGS). We hypothesize to find a good diagnostic accuracy of HAS assessed with the new procedure and AUC values of ≥ 0.85 [16, 43] with a 95 % confidence interval not going below a moderately accurate level of 0.7 [16, 44]. The diagnostic accuracy will be comparable to that of the fall risk assessments TUG, SPPB and GS and has a better accuracy than HGS. Additionally we expect a high accuracy of HAS in recognizing fallers in parallel testing with TUG, SPPB, GS, and HGS [17].

#### Study design

Part II is a validity study with prospective data collection regarding the external criterion (fall history) and a cross-sectional design for the evaluation of the construct validity.

#### Participants in and exclusion criteria

 People already participating in the AFFIRM-CT study, and having signed informed consent for this part will be included. For part II, community-dwelling and hospitalized participants will be included according to the same inclusion and exclusion criteria as the participants of part I of this study.

#### Procedure

From January 2021 on, participant recruitment is following the same procedure as described in part I until the scheduling of the first appointment after participants signed the informed consent.

During the first appointment, the study nurse will assess the following fall risk related variables: fall history of the last 6 months [6], poly-medication, and participants’ physical activity level assessed with a physical activity tracker worn on the wrist over 7 days. Then a health care professional blinded for participants’ fall history will assess the following functional fall risk assessments: SPPB [24], TUG [38], usual GS [25], and HGS [40]. Afterward, the second assessor blinded to the test results of the functional fall risk assessments and the fall history of the participants will measure participants’ HAS according to the given description of the index test. This assessor will receive prior to the measurements, a short 10min instruction of the use of the dynamometer, similar, to what would be done in a clinical situation. The HAS of the left and right side will be evaluated either by starting with the randomly chosen right or left side. As described in part I, the MVIS will be measured by asking the participants to push as hard as possible with the dynamometer against the wall (3 times per leg with a break of 30 seconds between each trial). Then the measurements are repeated, this time by pushing the dynamometer as fast as possible against the wall (assessment of the RFG).

Once the hip abductors strength (index test) is evaluated, participants will be followed up for a 12 months period to assess their number of falls (reference test). Participants will be provided with fall diaries in which they are asked to write down their falls and the circumstances/reasons of the falls (intrinsic or extrinsic) and occurring injuries (e.g. fractures) every time such an event will happen. Participants’ will thoroughly be explained what the definition of a fall is and how to fill out the fall diary. A fall is defined as an event resulting in a person inadvertently coming to rest on the ground, floor, or other lower level [2]. Every 6 months, the study nurse will call participants to collect the fall information and provide coaching for the fall diaries if needed.

#### Data collection

 Data collection will be performed as soon as the participant officially gave their permission and signed the informed consent. Participants’ information and test results will be entered into the electronic case report form (e-CRF).

##### Socio-demographic data and fall risk assessment

As for part I, the reliability part, the socio-demographic data (age, sex, height, and weight) will be obtained by a phone call or from the electronic participant record of the AFFIRM-CT study. A variety of non-functional fall risk related variables for the description of our participants will be assessed. The fall history of the last 6 months and the MMSE [28] will be evaluated by the study nurse exactly as described in the data collection section of part I of this protocol. The number and names of the medication the participants take will be written down on the participant’s documents to get an indication of their poly-medication. Further, participants’ physical activity level, assessed with a physical activity tracker worn on the wrist over a time span of 7 days, and the use of walking aids/assistive devices will be registered in the e-CRF.

##### Functional fall risk assessments

Additionally to the SPPB [24] and usual GS [25, 26] assessed exactly as described in part I, the Timed up and Go test (TUG) [38, 45] will be assessed. The time to complete the test at the usual GS will be recorded in seconds [45]. The hand-grip strength will be evaluated with a Jamar dynamometer with participants sitting on a chair, their elbow in 90° flexion with the forearm resting in a neutral position on the arm of a chair and the wrist in 0–30° dorsiflexion [40]. The strength will be evaluated in kg [40].

##### Index and reference test assessment

A health care professional/assessor, blinded for the test results of the functional fall risk assessments and the fall history of the participants, will measure participants’ HAS following the testing procedure described for the index test. The strength values will be entered in the e-CRF.

During the follow-up phase of 12 months, participants will be asked to complete fall diaries as soon as they experienced a fall. The study nurse additionally will call participants every 6 months.

#### Sample size calculation

According to our previous findings of the AUC of hip abductors strength in a standing position, good AUC values (0.85) are expected [16, 43] with a 95 % confidence interval not going below 0.7 [16]. Using the statistical environment R [31] with a significance level of 5 % and a power of 80 % we need 31 positive and 104 negative cases for the evaluation of the diagnostic accuracy (criterion validity). Compensating for an expected drop-out rate of 20 % over the 12 months follow up period, we will have to include 169 participants. As the sample size calculation for the construct validity is smaller than for the criterion validity we base our study on the sample size for the criterion validity.

#### Data analysis

Data analyses will be performed with SPSS version 25 (IBM Corp. Released 2017. Armonk, NY: IBM Corp.). Descriptive statistics for the sample characteristics and the functional and non-functional fall related variables will be performed. For continuous variables, the mean and standard deviation (mean ± SD) will be presented.

The definition of the evaluation of the MVIS and RFG of the HAS is described in our previous article [15]. MVIS and RFG will be normalized to body weight [16].

For the construct validity, the correlations of the HAS (MVIS and RFG) with the functional fall risk assessments SPPB, TUG, GS, and HGS will be presented.

For the criterion validity, the AUC and the diagnostic values of HAS, TUG, SPPB, GS, and HGS, compared to the chosen external criterion, history of falls, and 12 months fall prediction (sensitivity, specificity, PPV, NPV, LR+, and LR-) as well as cut-off values will be presented. We will further calculate the net sensitivity and net specificity to present the additional use of HAS in a test battery with other fall risk assessments (SPPB, TUG, gait speed). Additionally we will calculate indices for calibration and discrimination.

#### Data handling and storage for part I and part II of the study

Study participation is voluntary. Handling in the signed informed consent is a requirement before data collection can start. The Swiss legislation will be followed for the research data management and personal data of the participants. All data of study participants will be encrypted by a code. Participants ID will consist of the three letters HAS (hip abductor strength) and a continuous number starting from 001 up to 202 (ex. HAS_001). The coding sheet will be stored separately from all study documents in a cupboard and on a secured server of our institution in a folder only accessible by the principal investigator.

Throughout the entire trial, strict confidentiality is ensured and only a few authorized persons will have access to the study data. Respecting the Swiss law on human research (Federal Act on Research involving Human Beings (HRA)) and its applicable ordinance ClinO KlinV OClin OSRUm, no data that may identify subjects will be provided. According to the local standards for studies with short duration and minimal risk, no monitoring is planned for the nested-in study. Once data collection and analyses are completed, the coding sheet will be destroyed and no link between participants’ names and the codes can be made anymore. The data of the study will be stored for a minimum of 10 years in the institutional archiving system Yareta, the Research Data Repository of Geneva’s Higher Education Institutions. Within the limits of the ethical approval, the encrypted data may be used for related future research questions.

### Ethics and dissemination

This trial will be carried out in accordance with Swiss legislation, the Declaration of Helsinki [46], and Good Clinical Practice guidelines. The protocol was submitted to and approved by the ethics committee of Geneva (Commission Cantonal d’éthique de la recherche sur l’être humain (CCER), BASEC No 2019 − 01327). The project leader is promptly notified if immediate safety and protective measures have to be taken during the conduct of the research project. If a serious event occurs, the research project will be interrupted, and the Ethics Committee notified. Part I and part II of this study is nested within the AFFIRM-CT study and will be registered in ClinicalTrials.gov. After data collection and analyses are completed, the data of part I and part II will each lead to publications in international peer-reviewed journals.

## Discussion

The detection of persons at risk of falls is a major public health concern and should have high priority. Unfortunately, the diagnostic accuracy of currently used functional tests (e.g. SPPB and TUG) to assess older persons’ fall risks remains moderate and additionally, those tests usually don’t reveal directly targetable parameters for the prevention or rehabilitation of persons at risk. Amongst others, this led to the development of the above-mentioned adapted measurement instrument for hip abductors strength of older persons aged 65 years and over. Understanding the construct of older persons’ fall risk might benefit from the additional assessment of HAS as it is evaluating a complementary aspect of falls [17]. To perform several tests either in parallel or serial testing increases the certainty of detecting as many persons at risk of falls (true positives or true negatives) as possible (increase in net sensitivity and/or net specificity). As we know that the diagnostic accuracy of currently used functional fall risk assessments is only moderate, we are interested to investigate if HAS could improve a reliable and valid fall risk detection by its use in a test battery. Compared to the currently used functional performance tests to detect the fall risk (e.g. SPPB, TUG) HAS might be a directly targetable fall-risk parameter, which could contribute to treatment goal setting and might also to a well-targeted treatment (to be proven in future studies).

As there are not yet any articles available using the parameter hip abductors strength in a closed chain position, we need to investigate its clinimetric properties before a wide implementation can be considered. We will investigate this in a population of older adults with no additional diseases known to increase the fall risk. However, many diseases are known to increase the fall risk (e.g. neurological diseases) and future studies might investigate the additional use of the assessment of hip abductors strength for the assessment and treatment of these persons when reliability and validity are proven and acceptable in this study.

Regarding the clinical use of the measurement device for the hip abductors, we are expecting that the measurements are easily feasible in a practical time frame for clinical use, that its reliability for intra- and inter-tester use is high and it shows a good validity for the construct “fall risk” compared to the other selected measures and the differentiation of fallers and non-fallers compared to fall history.

Following the STARD guidelines [37], the methodological aspects important for a diagnostic study were specifically taken care of in this study. First, the selection of the population will be performed consecutively and corresponds to the specific settings in which the HAS measure will be used. The in-and exclusion criteria lead to a representative sample of the target population. The data collection is defined before the index and reference test will be performed and the assessor of the index test will be blinded for the results of the reference test. The nested nature of this study will not bias the study results of either study (reliability and validity part as well as the AFFIRM-CT study) as for both studies no intervention is planned as well as the included population is in line with the population in which the measurement instrument is meant to be used. The aim of the AFFIRM-CT study is to develop and validate an integrative fragility fracture prediction model. The model will integrate fall risk, bone strength, and fall biomechanics to predict a subject’s risk of fracture. Successful development and proper validation of this integrative fragility fracture prediction model will provide a better estimation of an individual’s fall and fracture risk and will allow broader screening across the growing population aged ≥ 65 years. The advantage of our study nested within the AFFIRM-CT study is that we get access to a high number of participants and that we investigate in a subsequent step if a combination of functional fall risk assessments (SPPB, TUG, GS, HGS, and HAS) with more medical information (e.g. bone density, impact load) can be advantageous to identify older persons’ at risk of falling. The information about the validity of the HAS measurement, on the other hand, will add to the precision of the prediction model of the AFFIRM-CT study. The functional testing position and clinically easily applicable test of the HAS measurement, as well as the 12 months prospective assessment of the numbers of falls of our participants, is a further big advantage of our study.

The blinding of the persons assessing hip abductors strength, for participants’ fall history and functional fall risk assessment test results, will be performed with great care. It is important that the assessor cannot intentionally influence the verbal encouragement during the strength testing of fallers or non-fallers according to their fall history for example. Additionally, we randomize the order of the assessors in the reliability part to simulate a situation as close to practical situations as possible. However, the randomization of the testing order for the functional fall risk assessments as well as the assessment of the MVIS before the RFG is not possible due to practical reasons at the testing location and to the nested nature of the presented study. Therefore, we will statistically evaluate if learning effects occurred and carefully check that the resting time of 5 min between the functional tests will be respected. Further, the statistician will be blinded for the outcomes of fallers and non-fallers for the analyses. To our knowledge, our measurement device is the first instrument easily accessible and transportable for clinical practice that is measuring HAS in a closed chain position. During the development process, it was of great importance that the developed measurement device is easily applicable in the daily clinical life of health care professionals. With the collected information about feasibility, we will get insight if this goal was achieved and the clinimetric properties will show if the measurement of HAS in a closed chain position should be encouraged as a reliable and valid measure for fall risk assessment in older adults.

## Data Availability

Not applicable.

## Abbreviations

ABD: Hip abductor
AUC: Area under the curve
CCER: Commission Cantonal d’éthique de la recherche sur l’être humain (ethical commission)
CI: Confidence interval
e-CRF: Electronic case report form
GS: Gait speed
HAS: Hip abductor strength
HGS: Hand grip strength
LoA: Limits of agreement
LR+: Positive likelihood ratio
LR-: Negative likelihood ratio
MMSE: Mini Mental State Examination
MVIS: Maximum voluntary isometric strength
NL: Netherlands
NPV: Negative predictive value
NRS: Numerating rating scale
PI: Principal investigator
PPV: Positive predictive value
RFG: Rate of force generation
SNF: Swiss National Science Foundation
SPPB: Short Physical Performance Battery
STARD: Standard of reporting diagnostic accuracy studies
TUG: Timed Up and Go
